# Impact of a pre-school milk on nutrient status, intake and growth in children aged 3–5 years old: a 16-week randomized, parallel clinical study

**DOI:** 10.3389/fnut.2025.1680946

**Published:** 2025-11-21

**Authors:** Elvira Estorninos, Rachel B. Lawenko, Jowena D. Lebumfacil, J. Edgar Winston C. Posecion, Veron Catrisse Delos Reyes, Alison Petit-Jean, Delphine Egli, Keith V. Tanda, Imelda Agdeppa

**Affiliations:** 1 Las Pinas Doctors Hospital, Las Pinas, Philippines; 2 Wyeth Nutrition, Makati City, Philippines; 3 Nestle Philippines, Makati City, Philippines; 4 Nestlé Product Technology Center – Nutrition, Société des Produits Nestlé S.A., Vevey, Switzerland; 5 Global Institute of Professional Development and Excellence Ortigas Center, Pasig, Philippines

**Keywords:** growth, nutrient adequacy, pre-school milk, nutrition, vitamin A deficiency

## Abstract

**Background:**

Recent research suggests there is an increasing burden of malnutrition in the Philippines due to insufficient or deficient consumption of micronutrients and energy. This open-label, randomized, prospective clinical study examines the impact of the consumption of a pre-school milk for young children on micronutrient status, nutritional intake, and growth.

**Methods:**

Healthy, singleton, term Filipino children aged 3–4.5 years were randomized to receive a fortified pre-school milk product (*n* = 56; INT) or consume their habitual diet (*n* = 55; CTRL). The primary outcome was nutritional status of key micronutrients, including vitamins A, D, E, and zinc. Micronutrient status was assessed at baseline and after 16 weeks of intervention. Secondary endpoints in this study included anthropometric measurements, nutrient intakes, developmental milestones, incidence of illnesses, and medication use.

**Results:**

Mean age at baseline was approximately 44 months old. Almost all children (98%) were consuming fortified milk before being enrolled in the study. The primary outcome, micronutrient status did not significantly differ between INT and CTRL at baseline or after 16 weeks (*p* > 0.05). Mean nutrient intakes were higher in INT compared to CTRL and closer to the recommendations. A significant increases in weight and height from baseline to endline were observed in both groups. After 16 weeks, mean weight and BMI were significantly higher in INT vs. CTRL (*p* < 0.005) with mean weight-for-age, height-for-age and BMI-for-age z-scores tracked closer to the WHO median for the INT group. The total number of sick days was 162 in INT vs. 181 in CTRL.

**Conclusion:**

In this randomized, parallel clinical study, children consuming a pre-school milk for 16 weeks had improved nutrient intakes and increased rate of growth compared to a group of children consuming their habitual diet.

## 1 Introduction

Milk and other dairy products provide energy, protein, and micronutrients which support normal growth and development in children (1). When the body does not receive enough or receives too much (i.e., any imbalance) of vitamins and other nutrients that play a role in normal tissue and organ development, malnutrition can occur (2, 3). Measures of malnutrition include stunting (height-for-age), underweight (weight-for-age), and wasting (weight-for-height), defined as more than two standard deviations (SD) below the World Health Organization (WHO) Child Growth Standards median. Malnutrition is a major public health concern in the Philippines (4). The 2021 National Nutrition Survey data in the Philippines indicated that the prevalence of stunting is 26.6%, underweight is 19.7%, and wasting is 4.8% among children less than 5 years old (5). The same survey showed that rice and other cereal products contributed up to 47.1% of the dietary intakes of Filipino children aged 3–5 years old while milk and milk products, represented 9.7% of their diet suggesting there could be a high inadequacy of energy and micronutrients (5).

Specific micronutrient deficiencies can limit normal growth and development and compromise a child’s ability to fight infections and other illnesses; this is referred to as “hidden hunger” (6, 7). These deficiencies translate into lower energy levels, mental clarity, and overall capacity and could lead to worse educational outcomes, longer illness duration and severity, and increased risk from other health conditions in the long-term trajectory of the child (8–10). Given enough nutrients through dietary interventions, catch-up growth is possible. Nutritional interventions studies conducted among preschool populations have demonstrated improvements in anthropometry, and micronutrient intake (11–13). Thus, the primary objective of this prospective study is to compare the status of key micronutrients in young children given Pre-school milk compared to young children consuming their habitual pattern of diet and beverage intake. Primary endpoints include the status of vitamins A, C, D, E and zinc at 16 weeks. Secondary objectives include growth, dietary intake, pre-school milk acceptability, and safety.

## 2 Methods

### 2.1 Study design and population

This two arm, open-label, randomized, parallel prospective interventional study was conducted between July and October 2022 at the Las Pinas Doctors Hospital in the Philippines. Children between 3 and 4.5 years of age were enrolled if they met the following criteria: healthy, singleton, term infant (37–42 weeks of gestation); birth weight ≥2,500 g and ≤3,900 g; weight-for-age, height-for-age, weight-for-height, and body mass index (BMI) z-score within −2 to +1 standard deviations using WHO child growth standards; and overall good health with no history of underlying metabolic or chronic disease, congenital malformation, or any other condition which could interfere with the child’s ability to ingest food, the normal growth and development of the child, or the evaluation of the child. Exclusion criteria were: presence of chronic infectious, metabolic, genetic illness or other disease that impacts feeding or any outcome measures; child exhibited any clinical signs of potential micronutrient deficiencies (e.g., hemoglobin <100 g/L based on screening assessment, rickets based on physical exam); child had known or suspected cows’ milk protein intolerance/lactose intolerance/allergy or severe food allergies that impact diet; breast milk was used exclusively/mixed in place of all other milk, and/or milk alternatives at 3 years of age; child consumed supplement(s) of relevance to the study outcome relating diet to status, vitamins A, C, D, and E and zinc; and lastly, investigator deemed the child’s family could not be expected to comply with the protocol or study procedures. The ethics committee at Las Pinas Doctors Hospital approved the study and the study was registered on ClinicalTrials.gov (NCT05440604). Parent(s)/legally authorized representative provided informed consent prior to study enrollment. This study was conducted in compliance with the International Conference on Harmonization Guidelines for Good Clinical Practice and the Declaration of Helsinki.

### 2.2 Randomization, intervention, and study product

Each child was randomized to one of two feeding regimens: pre-school milk or habitual diet for 16 weeks. The pre-school milk is a commercially available milk-based fortified beverages that includes micronutrients along with appropriate levels of protein and other macro- and micro-nutrients in alignment with global and local nutrient recommendations for children aged 3–5 (Table 1).

**Table 1:** Profile of pre-school milk (approximately 39 g of powder with 210 mL drinking water served twice daily).

Nutrient	Unit	Amount per 2 servings	Recommended nutrient intakes	% daily value in 2 servings*
Energy	kcal	338	1,350	25%
Protein	g	11.4	22	52%
Carbohydrate	g	48	NS**	
Total fat	g	10	NS**	
Vitamin A	IU	998	**1,332**	70%
Vitamin D	IU	198	*200*	100%
Vitamin E	IU	8	*7.5*	106%
Iron	mg	7.4	**9**	80%
Zinc	mg	3.8	**5**	80%
Vitamin C	mg	58	**45**	120%
Calcium	mg	476	**550**	80%

Recommended Nutrient Intakes (RNI) are in bold font, while Adequate Intakes (AI) are in italics. *Philippine Dietary Reference Intakes (PDRI) 2015: Summary Tables for 3–5 male age group. **Not stated.

The pre-school milk was placed in plain cartons containing 400 g with a clinical trial label that included preparation instructions. Children randomized to the pre-school milk intervention were instructed not to consume any other type of fortified milk and were encouraged not to replace the minimum recommended servings (2) of the pre-school milk with other milk or milk products. Investigational product dispensations were recorded in the case report form. The habitual diet consumed their regular diet and a habitual beverage intake pattern (e.g., milk and non-milk products including teas, juices, condensed milk, water, unfortified milk, and sugar-sweetened drinks). The majority (98%) of children in the habitual diet group continued to consume their habitual fortified milk thought the entire study. The study included a screening, clinic visits on day 1 (V1), week 8 (V2), and week 16 (V3), and phone calls at weeks 4 (C1) and 12 (C2).

Randomization was conducted by a statistician independent of the research team, using the ralloc package in Stata (14). Labeling of the treatments was also randomized and kept in a sealed envelope until after the data analysis was complete.

### 2.3 Nutrient biomarkers

Nutrient biomarkers were measured by the Department of Science and Technology-Food and Nutrition Research Institute, Taguig City, Philippines. A blood sample was obtained at V1 and V3 to measure the levels of key nutrients including vitamin A, vitamin D, vitamin E, and zinc. The primary efficacy assessment was the measurement of serum 25-hydroxy vitamin D [25 (OH) D] at V3 by chemiluminescent microparticle immunoassay (CMIA). Vitamin A and E was measured using serum retinol concentration while plasma alpha tocopherol was used to assess vitamin E by ultra-performance liquid chromatography (UPLC). Zinc status were measured by inductively coupled plasma/mass spectrometry (ICP/MS).

### 2.4 Dietary intake

A single 24-h dietary recall was conducted by a registered nutritionist-dietitians with the participant’s parent(s)/legally authorized representative using a structured questionnaire. The 24-h dietary recall was conducted at each visit (V1, V2, V3) and at phone calls (C1, C2). Only the interviewer recorded all foods and beverages consumed on the previous day from the moment the participant woke up until they went to sleep in the evening. The interviewer asked about measuring cup and spoon use to estimate total food and beverage intake. Energy and nutrient intakes were estimated using dietary computation software based on the Philippine Food Composition Table (PhilFCT^®^). The Philippine Dietary Reference Intakes (PDRI, 2015), which include the recommended nutrient intakes (RNI), adequate intakes (AI), and estimated average requirements (EAR), were used to evaluate nutrient adequacy.

Nutrient intakes of key interest included immunity-related nutrients (iron, zinc, selenium, vitamins A, C, and D) and nutrients for which intakes are known to be globally or locally insufficient or deficient (fiber, calcium, total fat, and protein). Additionally, a semi-quantitative food frequency questionnaire was administered at V1 and V3 to document the habitual pattern of food and beverage intake. Macro- and micronutrient intake (mean, percentage DRI) was calculated at V1, C1, V2, C2, and V3 using the 24-h dietary recall.

### 2.5 Compliance

Pre-school milk intake was closely monitored throughout the trial using the Milk Intake Diary and Study Compliance Diary. With an expected study duration of 16 weeks and a recommended intake of 2 servings per day, at least 169 of 226 (75%) servings must have been consumed over the entire study duration for the child to be considered compliant. Instructions for completing the diary were provided to the parent(s)/legally authorized representative at the baseline visit.

### 2.6 Additional outcomes

Insufficiencies of nutrients were also assessed at V3. Prevalence of nutrient insufficiencies (as applicable) and deficiencies were determined according to the following definitions: vitamin D insufficiency = 30–50 nmol/L; vitamin D deficiency = <30 nmol/L; low vitamin A (serum retinol) = ≤0.70 umol/L; iron insufficiency = adjusted serum ferritin <20 ug/L and serum transferrin receptor ≥35 nmol/L; iron deficiency = unadjusted serum ferritin <12 ug/L and CRP < 5 mg/L or unadjusted serum ferritin <30 ug/L and CRP ≥ 5 mg/L; anemia = Hb < 110 g/L; iron deficiency anemia = Hb < 110 g/L with unadjusted serum ferritin <12 ug/L and CRP < 5 mg/L or Hb < 110 g/L and unadjusted serum ferritin <30 ug/L and CRP > =5 mg/L; and zinc deficiency = <65 ug/dl for blood samples collected in the morning and <57 ug/dl for blood samples collected in the afternoon.

### 2.7 Growth

Anthropometric measures including weight, standing height, and body mass index were obtained at V1 and V3, and head circumference was obtained at V1. Children were weighed without clothing or diaper on a calibrated electronic weighing scale and measurements were recorded to the nearest gram. Height was measured using a stadiometer (SECA 213, SECA GMBH & Co., Germany) to the nearest 0.1 cm. Head circumference was measured using a pediatric non-elastic tape measure to the nearest 0.1 cm. Using the WHO Child Growth Standards as the reference population (15), corresponding z-scores including weight-for-age, weight-for-height, height-for-age, BMI-for-age, and head-circumference-for-age were calculated.

### 2.8 Adverse events

Adverse events (AEs), both serious (SAE) and non-serious, were recorded throughout the study. The type of event, incidence, seriousness, severity, and relation to feeding were documented. Child illnesses and infections including lower respiratory tract infections, upper respiratory tract infections, otitis/ear infections, constipation, diarrhea, vomiting, and gastroenteritis were also summarized in addition to the usage of medications such as anti-vomit/regurgitation, anti-pyretic/pain, and antibiotics. Lastly, the number of days the child was absent from day care or pre-school due to illness was recorded.

### 2.9 Sample size

A sample size of 112 children was calculated based on an effect size of 0.75, a significance level (2-tail test) of 5%, and a power of 95%, with an additional 15% allowance for dropouts for the primary objective of vitamin D concentration (16, 17). Secondary outcomes such as changes in zinc, vitamins A, E, and C required a smaller sample size.

### 2.10 Statistical analysis

The intention-to-treat (ITT) set included all screened children and were analyzed according to treatment assignment. The full analysis set (FAS) included all children from the ITT with serum concentration measures at V1 and V3 of either vitamin D, zinc, or ferritin. The safety analysis set (SAF) included all children from the ITT and were analyzed according to the feeding received. The per protocol analysis (PP) included all children from the FAS who did not have any major protocol deviations, violations of inclusion or exclusion criteria, or any documented concomitant medication intakes impacting one of the three primary endpoints. The primary endpoints of vitamin D, zinc, and ferritin concentrations were conducted using the FAS and PP populations while secondary endpoints were evaluated on the ITT set. Safety endpoints were examined on the SAF population.

Descriptive statistics were calculated for all variables. Normality of distributions were evaluated using the Kolmogorov–Smirnov test. Comparisons of outcomes between two groups were carried out using the Student’s *t*-test or the Mann–Whitney U test. Correlations were evaluated using Pearson’s or Spearman’s correlation coefficients. The Chi Square test was used to compare categorical measures between groups. Differences were considered statistically significant at *α* < 0.05. Analyses were conducted using Stata version 13 (18).

## 3 Results

There were 56 and 55 children enrolled in the pre-school milk and habitual diet groups, respectively. At baseline, there were no significant differences between groups in demographics or household characteristics (Table 2). Children were of similar age, with comparable proportions of males and females in both groups. Almost all children across both groups received fortified milk before beginning the study.

**Table 2:** Baseline demographic and household characteristics.

Characteristic	Habitual diet, *n* = 55	Pre-school Milk, *n* = 56	*p*-value^1^
Infant demographics			
Age at baseline, months	44.5 (0.6)	44.9 (0.6)	0.694
Gestational age, weeks	38.7 (0.8)	38.8 (0.9)	0.694
Infant sex, % female	50.9%	57.1%	0.510
Asian, Tagalog, % yes	100%	100%	1.000
Type of delivery, % C-section	12.7%	16.1%	0.589
Received breast milk, % yes	100%	98.2%	0.315
Received fortified milk, % yes	98.2%	98.2%	0.990
Started complementary food, % yes	98.2%	100%	0.315
Household characteristics			
Daycare attendance, % No	50.5%	49.5%	0.616
Cared outside home, % No	81.8%	83.9%	0.768
Maternal education, % completed beyond high school	30.9%	32.1%	0.983
Mother smokes, % no	90.9%	80.4%	0.105

Data presented are mean (SD) unless otherwise specified. ^1^*P*-value is resulting from two-sided test of difference between the habitual diet and pre-school milk groups.

### 3.1 Micronutrient status

Mean or median micronutrient status (vitamins A, D, E, and zinc) did not differ across the two groups at baseline or after 16 weeks (*p* > 0.05) (Table 3). Prevalence of vitamin A, D, E, and zinc deficiency did not differ significantly across the two groups at either baseline or after 16 weeks of intervention (Table 4). Majority of the children had normal levels of vitamin A (0.20–0.50 mg/L) in both the habitual diet (75%) and the pre-school milk (67%) at baseline and by the end of the study, while not statistically significant, a higher number of children had levels within the normal range (Habitual diet: 83%; Pre-school milk group: 80%) (Table 4). In contrast, most of the children displayed a vitamin D deficiency (<30 ng/mL) in both the habitual diet (58%) and the pre-school milk group (54%) at baseline and remain stable at endline in the habitual diet group while deficiency increased slightly (not statistically significant) to 64% in the pre-school milk group by the end of the study. Vitamin E was normal (>9 mg/L) for most of the children in both the habitual diet (89%) and the pre-school milk groups (92%) though the number of children with normal levels of vitamin E did decrease (not statistically significant) to 86% in the pre-school milk group. For zinc, 46% of the habitual diet and 36% of the pre-school milk groups had normal zinc levels (8.5–16 μmol/L) at baseline. At endline, the percentage of children that had normal zinc levels increased (not statistically significant) to 64 and 53%, respectively.

**Table 3:** Mean and/or median serum micronutrient status of vitamin A, vitamin D, vitamin E, and zinc.

Time point	Habitual diet, *n* = 52	Pre-school milk, *n* = 49	*P*-value^1^
Vitamin A, mg/L^‡^			
Baseline	0.46 (0.15)	0.44 (0.15)	0.923
Endline	0.41 (0.15)	0.40 (0.17)	0.892
	Habitual diet, *n* = 52	Pre-school Milk, *n* = 50	*P*-value^1^
Vitamin D, ng/mL^†^			
Baseline	29.7 (6.8)	29.3 (7.9)	0.784
Endline	29.2 (7.5)	27.7 (6.0)	0.288
	Habitual diet, *n* = 52	Pre-school Milk, *n* = 49	*P*-value^1^
Vitamin E, mg/L^‡^			
Baseline	12.1 (3.5)	12.3 (3.4)	0.660
Endline	11.8 (3.6)	12 (5.1)	0.116
	Habitual diet, *n* = 50	Pre-school milk, *n* = 47	*P*-value^1^
Zinc, umol/L^‡^			
Baseline	16.3 (3.4)	16.4 (2.7)	0.915
Endline	15.6 (5.7)	15.3 (5.7)	0.899

^†^Data presented are mean (SD), ‡ data presented are median (IQR) ^1^*P*-value is resulting from two-sided test of difference between the habitual diet and pre-school milk groups. ^2^*P*-value with the mean difference represents the change from baseline to endline within the group. Baseline was measured at clinical visit 1 (V1) and endline was measured at clinical visit 3 (V3).

**Table 4:** Distribution of micronutrient status of vitamin A, vitamin D, vitamin E, and zinc.

Time point	Habitual diet (*n* = 52)	Pre-school milk (*n* = 49)	*p*-value^1^
Vitamin A status, mg/L			
Baseline			
0.20–0.50	39 (75.0)	33 (67.4)	0.396
>0.50	13 (25.0)	16 (32.7)	
Endline			
0.20–0.50	43 (82.7)	39 (79.6)	0.514
>0.50	8 (15.4)	10 (20.4)	
*p*-value^2^	0.302	0.238	
	Habitual Diet (*n* = 52)	Pre-school Milk (*n* = 50)	*p*-value^1^
Vitamin D status, ng/mL			
Baseline			
<30	30 (57.7)	27 (54.0)	0.724
30–40	19 (36.5)	18 (36.0)	
Endline			
<30	27 (51.9)	32 (64.0)	0.131
30–40	19 (36.5)	17 (34.0)	
*p*-value^2^	0.727	0.388	
	Habitual diet (*n* = 52)	Pre-school milk (*n* = 49)	*p*-value^1^
Vitamin E status, mg/L			
Baseline			
3–9	6 (11.5)	4 (8.2)	0.570
>9	46 (88.5)	45 (91.8)	
Endline			
3–9	5 (9.6)	7 (14.3)	0.468
>9	47 (90.4)	42 (85.7)	
pvalue^2^	0.989	0.508	
	Habitual diet (*n* = 50)	Pre-school milk (*n* = 47)	*p*-value^1^
Zinc status, *umol/L*			
Baseline			
8.5–16	23 (46.0)	17 (36.2)	0.326
>16	27 (54.0)	30 (63.8)	
Endline			
8.5–16	32 (64.0)	25 (53.2)	0.280
>16	18 (36.0)	22 (46.8)	
*p*-value^2^	0.108	0.134	

Data presented are frequency (%) unless otherwise specified. ^1^Chi-square test, ^2^Mcnemar test*Significant at *p* < 0.05. Baseline was measured at clinical visit 1 (V1) and endline was measured at clinical visit 3 (V3).

### 3.2 Nutrient intake

Energy intake was within the recommended intakes for both groups but significantly higher in the pre-school milk group (Table 5; Supplementary Table 1). Higher mean intakes for dietary fat, iron, niacin, vitamin C, vitamin A, vitamin E, vitamin D, vitamin B12, folate, potassium, and zinc were observed in the pre-school milk group at endline. In contrast, the habitual diet group, consuming fortified milk, was not meeting requirements for energy, iron, vitamin D, or vitamin E. Moreover, by the end of the study, neither of the groups met nutrient intake requirements for dietary fibers or potassium.

**Table 5:** Mean macronutrient and micronutrient intake of children aged 3–5 years old by group.

Nutrient	Habitual diet		Pre-school milk		*p*-value^1^
	Baseline	Endline	Baseline	Endline	
Energy, kcal	1264.8 (598.9)	1113.5 (730.7)	1264.5 (561.7)	1217.4 (419.7)	0.051
Protein, g	41.2 (17.6)	31.8 (20.9)	38.9 (21.4)	37.5 (17.0)	0.089
Carbohydrates, g	181.4 (89.1)	164.5 (89.9)	192.4 (84.9)	192.6 (75.8)	0.066
Fat, g	42.6 (27.4)	28.8 (32.6)	43.8 (33.9)	43.6 (17.6)	**0.008**
Iron, mg	8.5 (5.4)	6.3 (5.1)	7.3 (7.9)	12.3 (3.9)	**<0.001**
Calcium, mg	611.4 (492)	373.8 (502.4)	638.3 (605.0)	629.0 (90.6)	0.050
Vitamin A, ugRE	220.9 (394.9)	112.3 (312.0)	229.2 (295.5)	402.8 (192.9)	**0.001**
Vitamin D, mg	2.4 (2.7)	1.6 (2.8)	2.5 (4.2)	5.8 (1.8)	**<0.001**
Vitamin E, mg	4.6 (3.8)	3.1 (3.0)	3.8 (4.9)	6.9 (1.8)	**<0.001**
Zinc, mg	5.0 (3.6)	3.93 (3.5)	4.8 (3.1)	6.4 (2.1)	**<0.001**
Selenium, μg	55.5 (39.8)	43.3 (34.3)	49.6 (41.3)	62.1 (42.5)	**<0.002**

Data presented are median (IQR) unless otherwise specified.^1^*P*-value is resulting from two-sided test of difference between the habitual diet and pre-school milk groups at endline. Baseline was measured at clinical visit 1 (V1) and endline was measured at clinical visit 3 (V3). Bold values represent statistically significant results.

At baseline the percentage of children falling below the thresholds of adequate nutrient intake was similar between the two groups. The percentage of children not reaching nutrient adequacy was greater than 40% for energy, vitamin A, vitamin D, vitamin E and iron. At end of the intervention, there was no change in the percent of children achieving nutrient adequacy in the habitual diet group for energy, protein, vitamin A, vitamin D, thiamine, vitamin B6, vitamin C, niacin, sodium, potassium or selenium while the percentage decreased for vitamin E, riboflavin, vitamin B12, calcium, phosphorous, magnesium, iron, and zinc by the end of the study compared to baseline. In the pre-school milk group, the percentage of children achieving nutrient adequacy for energy, protein, magnesium, sodium, potassium, and selenium did not change from baseline to the end of the study. At the end of the study the proportion of children achieving adequate intake in the pre-school milk group had significantly improved for vitamin A (*p* < 0.001), vitamin D (*p* < 0.001), vitamin E (*p* < 0.001), thiamine (*p* = 0.001), riboflavin (*p* = 0.016), vitamin B6 (*p* < 0.001), vitamin B12 (*p* = 0.001), vitamin C (*p* < 0.001), niacin (*p* = 0.001), calcium (*p* < 0.001), phosphorous (*p* = 0.031), iron (*p* < 0.001), and zinc (*p* < 0.001) compared to baseline intakes. Between groups, significant differences were detected in the percentage of children reaching nutrient adequacy following the 16-week intervention. Particularly, only 34.6% of children in the habitual diet group achieved vitamin A adequacy while 100% of the children in the pre-school milk group achieved nutrient intake adequacy (*p* = 0.001). Similar results were observed for iron and zinc, with only 32.7 and 63.6% of children achieving adequacy, respectively. In contrast, 100% of children in the pre-school milk group achieved adequacy for not only iron and zinc but also vitamin E, vitamin C, thiamine, riboflavin, vitamin B6, vitamin B12, niacin, and calcium.

### 3.3 Growth

At the beginning of the study, children were within normal but at the lower end of the WHO medians for weight, height, and BMI. Significant increases in weight and height were also observed within each study group from baseline to endline (Table 6). The rate of weight gain and height gain was 5 and 2%, respectively, in the habitual diet group, while in the pre-school milk group, the rate of weight and height was 9.2 and 2.4% at study end. At the end of the study, mean weight and BMI were significantly higher in the pre-school milk group compared to those maintaining their habitual diet (*p* = 0.005) with mean z-scores for weight-for-age, height-for-age, and BMI-for-age were closer to the median of WHO growth standards by the end of the study with a more pronounced effect among the children receiving pre-school milk. While all children included in the study were within normal, the pre-school milk group had a greater change in growth and moved closer to the WHO median.

**Table 6:** Mean anthropometric measurements and corresponding z-scores.

Time point	Habitual diet, *n* = 55	Pre-school milk, *n* = 56	*p*-value^1^
Weight, kg^†^			
Baseline	13.9 (1.3)	14.2 (1.5)	0.224
Endline	14.6 (1.3)	15.4 (1.8)	**0.005**
Mean difference (*p*-value)^2^	**0.7 (<0.001)**	**1.3 (<0.001)**	**<0.001**
Height, cm^†^			
Baseline	96.5 (3.2)	97.1 (4.3)	0.354
Endline	98.5 (3.3)	99.4 (4.4)	0.217
Mean difference (*p*-value)^2^	**2.0 (<0.001)**	**2.4 (<0.001)**	**0.068**
Body mass index (BMI)^†^, kg/m^2^			
Baseline	15.0 (0.9)	15.1 (0.9)	0.521
Endline	15.1 (0.9)	15.6 (1.1)	**0.005**
Mean difference (*p*-value)^2^	0.11 (0.179)	**0.56 (<0.001)**	**<0.001**
Weight-for-age z-scores^†^			
Baseline	−0.9 (0.7)	−0.8 (0.7)	0.358
Endline	−0.5 (0.7)	−0.1 (0.7)	**0.005**
Mean difference (*p*-value)^2^	**0.38 (<0.001)**	0.64 (<0.001)*	<0.001*
Height-for-age z-scores^†^			
Baseline	−1.1 (0.7)	−1.0 (0.7)	0.550
Endline	−0.6 (0.8)	−0.4 (0.7)	0.295
Mean difference (*p*-value)^2^	**0.50 (<0.001)**	**0.57 (<0.001)**	0.096
BMI-for-age z-scores^†^			
Baseline	−0.3 (0.7)	−0.2 (0.7)	0.471
Endline	−0.3 (0.7)	0.2 (0.8)	**0.004**
Mean difference (*p*-value)^2^	0.08 (0.209)	**0.4 (<0.001)**	**0.001**

†Data presented are mean (SD), ‡ data presented are median (IQR).^1^P-value is resulting from two-sided test of difference between the habitual diet and pre-school milk groups. ^2^*P*-value with the mean difference represents the change from baseline to endline within the randomized group. Baseline was measured at clinical visit 1 (V1) and endline was measured at clinical visit 3 (V3).Bold values represent statistically significant results.

### 3.4 Illness and infection

There were 28 and 44 reported AEs in the pre-school milk and habitual diet groups, respectively. More than 60% of these AEs were classified as “respiratory illness” which includes upper respiratory tract infections, lower respiratory tract infections, and similar illnesses (e.g., tonsilitis, allergic cough). During the 16-week intervention a total of 6,272 and 6,160 possible sick days were calculated for each group. Of those possible sick days 162 days were reported as actual sick days in the pre-school milk group and 181 days were reported in the habitual diet group. The pre-school milk group had in total a lower number of sick days by 11.8% as compared to those in the habitual diet group.

## 4 Discussion

In this randomized, parallel clinical study, children consuming pre-school milk for 16 weeks had improved nutrient intakes and growth compared to a group of children consuming their habitual diet which included fortified foods. Despite a low recorded dietary intake of vitamin A, vitamin D and vitamin E, most children at baseline had a normal micronutrient status for vitamin A and E. Vitamin D sufficiency, while not significative, decreased overtime in the pre-school milk group. Significant differences in nutrient status adequacy at both baseline and at the end of study was not observed between groups. This may be due to both groups were receiving popular fortified milk before enrollment considering the lipid-soluble nature of the individual vitamins measured (19). Serum retinol concentration are tightly regulated by the liver and does not reliably reflect liver vitamin A stores. Studies have shown that in children with adequate liver reserves, vitamin A supplementation does not improve serum retinol or liver stores (20, 21). Serum 25 (OH) D and alpha-tocopherol in children are typically sensitive to vitamin D and vitamin E intake from fortified foods even in situations of nutrient status adequacy (22) however we did not find any differences in serum status between groups in the current study despite an increase in reported intake. Serum zinc levels are consistent with results in literature which found no difference in serum or plasma zinc levels in participants consuming foods fortified with zinc plus other micronutrients when compared with participants consuming the same foods with micronutrients but no added zinc (23).

Interventional studies have shown that growing-up milk can contribute to overall nutrient intake and diet quality in young children. Effects of growing-up milks in children have reported improvements in weight, height, and body composition compared to milk with high protein or cow’s milk (11, 24, 25). At the end of the 16-week intervention period the pre-school milk supported a normal gain in height similar to the habitual diet group. Height has been shown to be improved by fortified milk supplementation over a 12-month intervention by others however the majority of studies under 12 months of intervention have not reported significant improvements in height (25, 26). A limitation to the current study was the short intervention period aimed at capturing early changes in micronutrient status, and a longer intervention period may be necessary to observe the impact of nutritional interventions on height in at risk populations (24). Milk fortified with specific micronutrients in a large population-based study showed a significant reduction in burden of common morbidities among preschool children, particularly respiratory tract infection (27). This may be due in part to an increase in intake of micronutrients including vitamin A and zinc. Interventional studies of micronutrient supplementation, specifically zinc (10 mg) combined with vitamin A (single dose) have reported a reduction in the percentage of days with upper respiratory tract infection in a population of preschool children with marginal nutritional status however serum status, after intervention, was not reported by the authors (28). In similarity, the current study which provided 5 mg of zinc daily and daily vitamin A as part of a pre-school milk, also reports a reduction in infection episodes, including respiratory tract infections and number of sick days.

Strengths of this study include the high completion rate which lowers the potential for bias from missing data and losses to follow-up. Further, standard techniques were utilized to assess nutrient intake and anthropometry thereby increasing the validity of the study findings. A main study limitation, is the short duration of the study as it is possible that at least 24 weeks are needed to observe meaningful improvements in micronutrient status height. Additionally, based on the 24-h dietary recall used in this study, almost all children in the habitual diet group consumed fortified milk for the general population. While this is reflective of the children’s habitual and local diet, this may have limited our ability to see differences related to micronutrient status in the comparative group. Zinc intake has been inconsistently associated with serum zinc concentrations in school-aged children, as reported by Bui et al. (29). This variability may stem from several methodological and biological factors, including the duration and consistency of zinc intake, as well as the presence of dietary inhibitors such as phytic acid, which can impair zinc absorption. In the current study, dietary intake was assessed using a single 24-h recall conducted by a trained dietitian based on parental reporting. In contrast, other studies employed more robust dietary assessment methods, such as three non-consecutive 24-h recalls (including two weekdays and one weekend day), which provide a more representative snapshot of habitual intake.

Awasthi et al. (30) demonstrated that food frequency questionnaires (FFQs) are effective in capturing long-term dietary patterns and micronutrient intake. Their study found significant associations between dietary intake and biochemical deficiencies of iron, vitamin A, and zinc—associations that were not observed in the present analysis. Notably, the current study did not account for phytic acid intake, a known anti-nutritional factor that inhibits the bioavailability of both zinc and iron. This omission may partly explain the drop in serum zinc status despite improved intake, whereas studies that considered phytic acid intake, reported a positive association (31).

In this randomized, parallel clinical study, children consuming a pre-school milk for 16 weeks did not improve nutritional status in the blood. Consuming pre-school milk did improve nutrient intakes and supported normal growth. Findings from this study are limited by the short intervention duration and prevalence of fortified milk use in the study population. In conclusion, this study reports that replacing fortified milk in the diet with an age adapted pre-school milk product increased micronutrient intake adequacy but not nutritional status of pre-school children with normal nutritional status and weight.

## Data Availability

The raw data supporting the conclusions of this article will be made available by the authors, without undue reservation.
