# Supplementary material for: Impact of a pre-school milk on nutrient status, intake and growth in children aged 3–5 years old: a 16-week randomized, parallel clinical study
Source: Front Nutr. 2025 Nov 21;12:1680946. doi: 10.3389/fnut.2025.1680946 (PMC12679889; doi:10.3389/fnut.2025.1680946)
Supplement: Supplementary file 1 [file Table_1.docx]

**Supplementary Table 1**. Mean intake of remaining macro and micronutrients of children aged 3-5 years old by group

| **Nutrient** | **Habitual Diet** | | **Fortified Milk** | |
| --- | --- | --- | --- | --- |
|  | **Baseline** | **Endline** | **Baseline** | **Endline** |
| Sfa, g | 14.4 (9.4) | 16.6 (38.0) | 14.1 (9.7) | 17.6 (30.4) |
| Mufa, g | 9.2 (5.9) | 8.3 (10.2) | 9.1 (6.5) | 10.6 (9.4) |
| Pufa, g | 4.7 (3.9) | 5.3 (6.0) | 4.4 (3.5) | 6.7 (4.9) |
| Thiamin, mg | 0.9 (0.6) | 0.8 (0.7) | 0.9 (0.6) | 1.0 (0.3) |
| Riboflavin, mg | 1.0 (0.3) | 0.9 (0.7) | 1.2 (0.7) | 1.1 (0.2) |
| Niacin, mg | 11.9 (19.8) | 9.1 (6.8) | 9.7 (5.7) | 13.5 (3.0) |
| Vitamin C, mg | 33.3 (32.4) | 26.5 (29.0) | 29.3 (30.8) | 72.2 (21.3) |
| Vitamin B6, mg | 0.7 (0.4) | 0.7 (0.4) | 0.8 (0.4) | 1.1 (0.3) |
| Vitamin B12, mg | 1.7 (1.1) | 1.3 (1.1) | 2.0 (1.2) | 2.8 (1.1) |
| Folate, ugdfe | 229.8 (137.4) | 196.2 (141.6) | 246.5 (199.3) | 275.7 (101.7) |
| Dietary fiber, g | 6.0 (3.6) | 5.3 (3.6) | 5.9 (3.7) | 4.7 (2.9) |
| Total sugar, g | 40.9 (33.4) | 31.6 (26.0) | 39.6 (29.1) | 23.4 (20.8) |
| Sodium, mg | 976.9 (481.6) | 879.8 (730.1) | 1009.2 (526.7) | 935.5 (564.7) |
| Phosphorus, mg | 755.4 (325.5) | 639.9 (340.1) | 789.3 (387.8) | 711.1 (148.0) |
| Magnesium, mg | 125.7 (61.7) | 102.8 (55.7) | 120.8 (46.1) | 125.2 (31.2) |
| Potassium, mg | 1089.8 (539.2) | 888.6 (539.1) | 1144.7 (563.5) | 1219.1 (296.6) |

Data presented are mean (SD) unless otherwise specified.
